# Chief Resident Skills: A Study on Resident Perceptions of Skill Importance and Confidence

**DOI:** 10.51894/001c.6466

**Published:** 2018-04-27

**Authors:** Tonya VanOrder, Samuel J. Wisniewski

**Affiliations:** 1 College of Osteopathic Medicine Statewide Campus System Michigan State University College of Osteopathic Medicine, East Lansing, MI

**Keywords:** gender, skills confidence, skills importance, chief resident skills

## Abstract

**CONTEXT:**

Chief residents (CRs) generally play a pivotal role in the graduate medical education mission to facilitate the professional development of resident physicians. Courses designed to prepare CRs for their new role previously have primarily focused on developing their teaching, evaluation and interpersonal communication skills. What remains unclear is how different types of residents (CRs versus Non-CRs and men versus women) may vary in their perception of how important particular skills are, and their confidence in performing these same skills. The purpose of this cross-sectional descriptive correlational study was to investigate the potential differences in sample respondents’ perceived importance of CR skills and respondents’ perceived confidence to perform these skills.

**METHODS:**

The authors administered a 28-item survey questionnaire to a population of 457 CR and Non-CR respondents from 20 clinical specialties regarding their perceived importance of 11 skills, as well as their self-assessed confidence in performing each skill. This study also sought to examine whether gender-based differences existed for the perceived importance and confidence of these same CR skills.

**RESULTS:**

Statistically significant differences in perceived importance and confidence levels between sample subgroups for the majority of key CR skills were observed. When asked about importance of CR skills, both CR and Non-CR respondents identified administrative and time management skills as most important, contrary to the other types of skills (e.g., teaching skills) the authors had generally expected to be most highly rated. As expected, the largest overall gap in perceived importance and self-reported confidence as either a CR or Non-CR was in the area of conflict management. Males reported higher confidence than females in each of the CR skills, with differences for five items found to be statistically significant. (p < 0.05) CR respondents also reported higher confidence in performing 10 of 11 skills at statistically significant levels. (p < 0.05)

**CONCLUSIONS:**

Although this project contributes baseline data from a relatively large sample, further studies are still required to replicate these results in other resident populations to further examine the perceptions of contemporary resident physicians concerning this vital role.

## INTRODUCTION

Today, resident physicians must develop a multitude of medical skills while providing safe patient care.^1–3^ Residents must acquire critical non-cognitive skills involving interpersonal communication, mitigating conflicts, team leadership, and teaching.^4–6^ Some residents may serve in a more involved role as chief residents (CRs) during the final year of training or for a one-year post-residency term. The CR role specifically entails leadership and administrative responsibilities in addition to traditional teaching/supervisory activities with Non-Chief residents (Non-CRs).^2,5^

Additionally, the dual nature of a CR as both leader and learner adds complexity during interactions with faculty, students and patients. Difficulties in communication or performance through the exercise of various roles can arise if the CR does not possess appropriate skills, knowledge, and abilities to carry out their role.^7^ Poor CR leadership can have a negative influence on Non-CRs, increasing conflicts within care teams, and possibly imposing adverse effects on patient care due to communication and coordination issues.^8^

The graduate medical education (GME) literature describes the importance of CR skills for teaching Non-CRs and medical students, leading and organizing teams, and managing administrative program affairs.^9–11^ However, not all CRs enter the role with the necessary skills and confidence to be effective leaders.^8,9^ Over the past two decades, CR training courses have become a popular way of providing additional preparation for this critical role.^8,12,13^

What remains inadequately understood is whether CRs or Non-CRs would identify similar CR skills and behaviors as being critically important, or how confident they may feel in demonstrating those skills themselves. In addition, gender differences in CR leadership approaches and outcomes have apparently not been studied in the GME literature. In other fields such as business management, wherein leadership theory and practice have been studied extensively, some scholars have suggested practical differences between management approaches of male and female leaders exist to influence business outcomes.^14–17^ As such, the authors also wished to examine whether gender might significantly influence self-reported sample levels of CR skill importance or confidence in this similarly leadership-oriented role.

In examining the historical context of how CRs typically obtain leadership knowledge, CRs have traditionally learned important leadership skills primarily through independent means. They have typically done this by observing and emulating attending faculty members, using trial and error, or at best, by receiving cognitive apprenticeship mentoring.^4,5,18^ More recently, formal CR training courses have typically included content concerning CR behaviors and expectations specifically deemed important by clinical faculty.^19^

Although the insights of current GME faculty are critical when designing effective CR course content, adult learning theory asserts that when adult learners can provide input into curricular design, their learning can be more effective and sustainable.^20,21^ Since Non-CR learners are important stakeholders in GME processes, it is critical that systematic assessments of their perceived training needs are incoporated.^20–22^

### Purpose of Study

The purpose of this cross-sectional descriptive correlational study was to investigate the correlations between respondents’ perceived importance of CR skills and their personal confidence in performing each skill. Using a modified 28-item quantitative survey, the authors examined whether they could identify any gender or CR vs. Non-CR subgroup differences in survey items. The authors’ overall null hypothesis was that they would be unable to identify and perceived differences when stratifying responses by either gender or CR-Non-CR status.

## METHODS

### Sample Participants

After campus-based IRB approval in 2017, the authors administered the confidential study survey to a convenience sample of all residents (n = 1,986) currently enrolled in residency training programs affiliated with the Michigan State University Statewide Campus System (SCS) GME consortium.^23^ Those physicians who had already graduated from a residency program were excluded.

All SCS-affiliated residents comprised the population of interest. This study was not limited only to CRs, since the authors also wanted to understand the perceptions of Non-CRs concerning the role. Prospective respondents were from 20 clinical specialties (e.g., family medicine, orthopedic surgery, neurology, OB-GYN, emergency medicine) across 35 different hospital locations throughout Michigan. The demographic data that were collected included year in GME training (PGY year), gender, clinical specialty, approximate program size, and whether the respondent was a current CR or not.

### Materials & Procedures

All data were obtained through a non-validated online Survey Monkey survey questionnaire instrument.^24^ (see Appendix 1 for survey instrument) Survey questions had been initially developed and content validated by a team composed of Ms. VanOrder with a GME faculty physician (Dr. Saroj Misra, FM Program Director) and the second author (SW). No existing instruments had been identified to assess CR skills, abilities, and behaviors from the Non-CR perspective. However, the format of the modified questionnaire utilized here was adapted from an instrument developed by the Sustainable Management Development Program of the Centers for Disease Control and Prevention (CDC/SMDP).^25^

The second part of the CDC/SMDP questionnaire asked public health employees 22 questions about core management competencies. For each competency, two components were surveyed: individual perception of the importance of the behavior (skill importance), and perceived level of knowledge/skills to perform that particular behavior (skill confidence).

Equally-weighted skill importance and confidence responses were operationalized as ordinal independent variable measures using a Likert-type scale of “1” to “5.” A rating of 1 indicated lowest perceived importance/confidence, and a rating of 5 indicates highest perceived importance/confidence. Similar to the CDC/SMDP survey,^25^ a five-point Likert scale was used to ask participants to rate the perceived level of perceived importance of 11 CR skills, and also as a range for level of confidence to perform each skill. The authors grouped questions as follows; four questions about CR leadership skills, four questions regarding CR teaching skills, and three questions about CR administration and time management.

Survey responses were aggregated by gender and current CR or Non-CR status. Statistical analysis examined the presence of a negative or positive correlation (Pearson *r*) between skill importance and skill confidence responses. The presence of the possible moderator variable gender was included for any effect it may have had imposed any relationship between perceived skill importance and skill confidence level responses. The second author (SW) conducted all analytic procedures using SPSS version 25 analytic software.^26^

## RESULTS

### Descriptive Statistics

A total of 457 (23% of 1,986 SCS-affiliated) residents responded, with 12 (0.6%) emails bouncing back to the authors and 20 (1.01%) residents opting to simply open up the survey program without completing any items. Respondents consisted of 116 (25.3%) CRs and 341 (74.5%) Non-CRs. A total of 389 (84.9%) respondents replied to every survey question, while 69 (14.1%) only partially completed the survey. 240 (52.4%) were male, and 204 (44.5%) were female, with the remaining 13 (3.1%) choosing not to specify their gender. There were approximately 421 (91.9%) D.O.’s and 21 (4.6%) M.D.’s, and the remaining 14 (3.5%) respondents did not specify their type of medical degree. Self-reported level of PGY experience included 12 (2.6%) Fellows, 103 (22.5%) PGY1s, 110 (24.0%) PGY2s, 116 (25.3%) PGY3s, 71 (15.5%) PGY4s, and 31 (6.8%) PGY5s. Fifteen respondents (3.3%) did not specify their residency year.

Base descriptive data analyses revealed that when ranking the importance items of the Non-CR subgroup, the results demonstrated a mean score range of 2.89 (SD = 1.10) (i.e., importance of leadership Quality Improvement (QI) projects) to 4.65 (SD = 0.76) (i.e., importance of managing conflict). Those three (out of 11) items ranked as most important by Non-CRs included: a) administrative responsibilities, b) time management and efficiency, and c) managing conflict. See Table 1 for results for all survey item responses from the non-CR subgroup regarding importance.

**Table 1:** Mean Non-Chief Resident Importance vs. Confidence Item Ratings (N = 341)

**Question:**	**Importance (SD)**	**Confidence (SD)**	**Difference**
*Lead health care teams*	3.89 (1.01)	2.95 (1.10)	0.94
*Lead quality Improvement*	2.89 (1.10)	2.41 (1.15)	0.48
*Lead communication*	3.84 (0.96)	**3.24 (1.01)**	0.6
*Lead wellbeing*	3.69 (1.03)	2.95 (1.03)	0.74
*Teach feedback*	4.07 (0.93)	2.97 (0.98)	**1.10**
*Teach plan*	3.77 (0.99)	3.07 (1.18)	0.70
*Teach skills*	4.06 (0.91)	**3.13 (1.05)**	0.93
*Teach effective*	3.71 (0.95)	2.89 (0.99)	0.82
*Conflict Management*	**4.65 (0.76)**	3.05 (1.01)	**1.60**
*Administrative responsibilities*	**4.15 (0.89)**	2.86 (1.14)	**1.29**
*Time Management and Efficiency*	**4.25 (0.78)**	**3.15 (1.08)**	**1.10**

*bolded are the top 3 highest in each column

When examining the mean score for importance for CR respondents, a similar spread from a lowest importance ranking for importance of leading QI projects, 2.97 (SD = 1.14) to that labeled as the highest importance, that of time management, at 4.41 (SD = 0.78). The three items ranked as most important by CRs included: a) teaching skills, b) managing conflict, and c) time management (See Table 2)

**Table 2:** Mean Chief Resident Importance vs. Confidence Ratings (N = 116)

**Questions:**	**Importance (SD)**	**Confidence (SD)**	**Difference**
*Lead health care teams*	3.85 (1.09)	3.61 (1.03)	0.24
*Lead quality improvement*	2.97 (1.14)	2.76 (1.08)	0.21
*Lead communication*	3.92 (0.98)	3.61 (0.97)	0.31
*Lead wellbeing*	3.63 (1.14)	3.18 (1.09)	0.45
*Teach feedback*	4.04 (1.01)	3.39 (0.97)	**0.65**
*Teach plan*	4.11 (0.91)	**3.92 (0.97)**	0.19
*Teach skills*	**4.16 (0.84)**	**3.81 (0.88)**	0.35
*Teach effective*	3.85 (0.95)	3.43 (0.97)	0.42
*Conflict Management*	**4.31 (0.81)**	3.35 (1.12)	**0.96**
*Administrative responsibilities*	4.13 (0.94)	3.44 (1.19)	**0.69**
*Time Management and Efficiency*	**4.41 (0.78)**	**3.77 (1.04)**	0.64

*bolded are the top 3 highest in each column

Pearson r correlation analyses were performed to investigate whether there were any statistically significant associations for each question variant asking about confidence and the paired question asking about importance (e.g., “How confident are you with your ability to lead multidisciplinary health care teams?” and “How important is leadership of multidisciplinary healthcare teams as a skill all chief residents need to have?”). Bivariate correlations were statistically significant (p < 0.05) for all 11 question pairs (8a and 8b -18a and 18b). (See Table 3)

**Table 3:** Associations between Importance and Confidence Ratings (N = 457)

**Confidence + Importance Correlation pairs**	**Pearson's r**	**p-value**
lead confidence + lead importance	0.377	< 0.05*
lead confidence QI + lead importance QI	0.506	< 0.05*
lead confidence communication + lead importance communication	0.511	< 0.05*
lead confidence wellbeing + lead importance wellbeing	0.41	< 0.05*
teach confidence feedback + teach importance feedback	0.406	< 0.05*
teach confidence plan + teach importance plan	0.478	< 0.05*
teach confidence skills + teach importance skills	0.47	< 0.05*
teach confidence effective + teach importance effective	0.43	< 0.05*
time man confidence conflict + time man importance conflict	0.158	< 0.05*
time man confidence admin + time man importance admin	0.381	< 0.05*
time man confidence time man + time man importance time man	0.423	< 0.05*

*statistically significant association between importance and confidence, p < 0.05

Inferential analytics were then conducted utilizing independent t tests to examine for differences between means for each question variant (importance and confidence) by gender. Statistically significant results (p < 0.05) were observed across the questions asking about perceived confidence for the following skills:

**Confidence of Skills by Gender**: (see Table 4 for full results)

**Table 4:** Mean Importance Ratings by Gender (N = 444)

**Question**	**Male (SD)**	**Female (SD)**
*lead importance*	3.82 (1.09)	3.96 (0.95)
*lead importance QI*	2.91 (1.08)	2.91 (1.14)
lead importance com*	**3.69 (1.00)**	**4.06 (0.88)**
lead importance wellbeing*	**3.47 (1.05)**	**3.92 (0.96)**
teach importance feedback*	**3.95 (0.99)**	**4.19 (0.84)**
*teach importance plan*	3.84 (1.00)	3.89 (0.97)
*teach importance skills*	4.02 (0.96)	4.18 (0.85)
teach importance effective*	**3.65 (0.98)**	**3.86 (0.89)**
*importance manage conflict*	3.98 (0.79)	4.36 (0.71)
importance admin*	**4.06 (0.95)**	**4.24 (0.82)**
*importance time management*	4.26 (0.84)	4.33 (0.70)

*statistically significant difference between male and female respondents, p < 0.05

Leadership

- Confidence, ability to lead:
  - Male (M) 3.26 (SD = 1.15) vs. Female (F) 2.98 (SD = 1.07)

Teaching

- Confidence, giving effective feedback:
  - M 3.25 (SD = 0.99) vs. F 2.89 (SD = 0.98)
- Confidence, skills in mentoring and coaching:
  - M 3.42 (SD = 1.05) vs. F 3.20 (SD = 1.04)
- Confidence, using effective teaching strategies and practices:
  - M 3.17 (SD = 1.02) vs. F 2.88 (SD =1.00)

Time Management

- Confidence, communication strategies for managing conflict:
  - M 3.26 M (SD = 1.05) vs. F 2.97 (SD = 1.03)

Similarly, statistically significant results (p < 0.05) were found between female and male respondents across the perceived importance questions:

**Importance of Skills by Gender**: (see Table 5 for full results)

**Table 5:** Mean Confidence Ratings by Gender (N = 444)

**Question**	**Male (SD)**	**Female (SD)**
lead confidence*	**3.26 (1.15)**	**2.98 (1.07)**
*lead confidence QI*	2.55 (1.15)	2.44 (1.14)
*lead confidence com*	3.34 (1.03)	3.34 (1.00)
*lead confidence wellbeing*	2.99 (1.04)	3.04 (1.07)
teach confidence feedback*	**3.24 (0.99)**	**2.89 (0.98)**
*teach confidence plan*	3.33 (1.18)	3.27 (1.19)
teach confidence skills*	**3.41 (1.05)**	**3.20 (1.04)**
teach confidence effective*	**3.17 (1.02)**	**2.88 (1.00)**
confidence manage conflict*	**3.26 (1.05)**	**2.97 (1.03)**
*confidence admin*	3.02 (1.17)	3.02 (1.09)
*confidence time management*	3.29 (1.12)	3.36 (1.09)

*statistically significant difference between male and female respondents, p < 0.05

Leadership

- Importance, understanding leadership and communication styles:
  - M 3.69 (SD = 1.00) vs. F 4.06 (SD = 0.88)
- Importance, facilitating well-being of self and others:
  - M 3.47 (SD = 1.05) vs. F 3.92 (SD = 0.96)

Teaching

- Importance, giving effective feedback:
  - M 3.95 (SD = 0.99) vs. F 4.19 (SD = 0.84)
- Importance, using effective teaching strategies and practices:
  - M 3.65 M (SD = 0.98) vs. F 3.86 (SD = 0.89)
- Importance, administrative management responsibilities:
  - M 4.06 (SD =0.95) vs. F 4.24 (SD = 0.82)

When examining confidence responses across CR-Non-CR status, statistically significant results (p < 0.05) were observed between the two groups.

**Confidence of Skills by CR-Non-CR status**: (see Table 6 for full results)

**Table 6:** Mean Confidence Ratings by Chief Resident Status (N = 116)

**Questions**	**Non Chief Resident (SD)**	**Chief Resident (SD)**
lead confidence*	**2.95 (1.10)**	**3.61 (1.03)**
lead confidence QI*	**2.40 (1.15)**	**2.76 (1.08)**
lead confidence com*	**3.24 (1.01)**	**3.61 (0.97)**
*lead confidence wellbeing*	2.95 (1.03)	3.18 (1.09)
teach confidence feedback*	**2.96 (0.98)**	**3.39 (0.97)**
teach confidence plan*	**3.07 (1.18)**	**3.92 (0.97)**
teach confidence skills*	**3.13 (1.05)**	**3.81 (0.88)**
teach confidence effective*	**2.88 (0.99)**	**3.43 (0.97)**
confidence manage conflict*	**3.03 (1.01)**	**3.35 (1.12)**
confidence admin*	**2.85 (1.14)**	**3.44 (1.19)**
confidence time management*	**3.15 (1.08)**	**3.77 (1.04)**

*statistically significant difference between non chief and chief residents, p < 0.05

Leadership

- Confidence, ability to lead:
  - Non-CR 2.95 (SD =1.10) vs. CR 3.61 (SD = 1.03)
- Confidence, developing Quality Improvement (QI) projects:
  - Non-CR 2.40 (SD = 1.15) vs. CR 2.76 (SD = 1.08)
- Confidence, understanding leadership and communication styles:
  - Non-CR 3.24 (SD = 1.01) vs. CR 3.61 (SD = 0.97)

Teaching

- Confidence, giving effective feedback:
  - Non-CR 2.96 (SD = 0.98) vs. CR 3.39 (SD =0.97)
- Confidence, planning and conducting educational sessions:
  - Non-CR 3.07 (SD = 1.18) vs. CR 3.92 (SD = 0.97)
- Confidence, skills in mentoring and coaching:
  - Non-CR 3.13 (SD =1.05) vs. CR 3.81 (SD =0.88)
- Confidence, using effective teaching strategies and practices:
  - Non-CR 2.88 (SD =0.99) vs. CR 3.43 (SD = 0.97)

Time Management

- Confidence, communication strategies for managing conflict:
  - Non-CR 3.03 (SD = 1.01) vs. CR 3.35 (SD = 1.12)
- Confidence, administrative management responsibilities:
  - Non-CR 2.85 (SD = 1.14) vs. CR 3.44 (SD = 1.19)
- Confidence, skills in time management and efficiency:
  - Non-CR 3.15 (SD =1.08) vs. CR 3.77 (SD =1.04)

When examining perceived importance responses across CR-Non-CR status, statistically significant results (p < 0.05) were found between the two groups for only one survey item:

**Importance of Skills by CR-Non-CR status**: (see Table 7 for full results)

**Table 7:** Mean Importance Ratings by Chief Resident Status (N = 341)

**Questions**	**Non Chief Resident (SD)**	**Chief Resident (SD)**
*lead importance*	3.89 (1.01)	3.85 (1.09)
*lead importance QI*	2.89 (1.10)	2.97 (1.14)
*lead importance com*	3.84 (0.96)	3.92 (0.98)
*lead importance wellbeing*	3.69 (1.03)	3.63 (1.14)
*teach importance feedback*	4.07 (0.93)	4.04 (1.01)
teach importance plan*	**3.77 (0.99)**	**4.11 (0.91)**
*teach importance skills*	4.06 (0.91)	4.16 (0.84)
*teach importance effective*	3.71 (0.95)	3.85 (0.95)
*importance manage conflict*	4.65 (0.76)	4.31 (0.81)
*importance admin*	4.15 (0.89)	4.13 (0.94)
*importance time management*	4.25 (0.78)	4.41 (0.78)

*statistically significant difference between non chief and chief residents, p < 0.05

Teaching

- Importance, planning and conducting educational sessions:
  - Non-CR 3.77 (SD = 0.99) vs. CR 4.11 (0.91)

## DISCUSSION

In summary, the authors found statistically significant differences in perceived importance and confidence levels between sample subgroups for the majority of key CR skills listed in the study survey. In terms of the perceived importance of specific CR skills, respondents identified administrative time management skills as most important, contrary to other types of skills (e.g., teaching skills) that the authors had generally expected to be rated as more important. However, as the authors had expected, the largest overall gap in perceived importance and self-reported confidence as a CR vs. Non-CR was in the area of conflict mitigation.

The authors had expected to find males to be more confident than females in most CR skills, and these results did demonstrate that sample men were overall more confident than women for each of the 11 survey skills, with differences for five items found to be statistically significant. (p < 0.05) This finding is similar to a previous study examining surgical resident self-assessment in which females were found to rate their overall skills and abilities (confidence) lower than men.^27^ As might be expected, we found CR respondents to be more confident than non-CR residents for 10 of the 11 survey items at statistically significant levels. (p < 0.05) This finding suggests that CR pre-survey experiences and/or prior CR training may have helped them increase their skill confidence.

These findings pose several intriguing questions for future GME studies. Further studies are needed to investigate how to meet the perceived learning needs of men vs. women and residents soon to assume CR positions. Targeted educational interventions to help newer CR develop non-medical care skills (e.g., administrative skills, leading QI projects, teaching strategies for resident peers, etc.) will require further testing. It remains unclear how such types of GME workshops/training could be customized for various clinical specialty areas, or between academic versus community-based learner groups.^4,8^

In addition, these findings appear to both match and contrast the results of earlier studies conducted to date. GME authors have predominately studied preferred CR role skills and abilities from the perspective of faculty and medical educators.^4,18^ In most GME settings, the most important CR skills are determined by clinical faculty based on: 1) accepted leadership characteristics, 2) core responsibilities to facilitate patient safety and quality care, and 3) CR role skills assumed to be most important.^4,8,18^ Few CR training courses have apparently been designed based on resident needs assessments.^7,20,28^

The results reported in this paper certainly indicate that it may be equally important to identify critical perceived CR skills from the perspective of resident learners themselves. Varied perceptions of both CR and Non-CR residents’ confidence levels and a better understanding of the potential influence of gender may help guide GME faculty to plan and modify effective CR training courses.

### Limitations

Since the authors used a cross-sectional quantitative survey questionnaire design with a convenience sample of Michigan residents, these findings may lack generalizability for many GME residency program environments. In addition, the presence of multiple potential sources of bias (e.g., self-selection, preferred responses, non-validated survey item wording) may have influenced the results.^28,29^ Future research designs could also entail more objective comparisons of actual CR performance vs. their perceived confidence in competently preforming skills. Finally, additional comparisons across tiers of PGY levels as well as between specialties and program size could also help explain the relationships between perceived CR skill importance and confidence. Further analyses utilizing the data collected here are being planned by the authors to investigate possible differences across PGY experience, clinical specialty resident groups and program size.

## CONCLUSIONS

Developing an improved understanding of the most critical CR skills and abilities has important implications for GME officials. First, further exploring self-identified CR training needs will allow for future comparisons of clinical faculty assessment ratings to inform their re-alignment of CR role expectations. Second, program directors and resident peers often select CR based on their limited demonstration of leadership and teaching qualities. Other skills or abilities (e.g., administrative management strategies, promotion of personal well-being, conflict management) might be equally or more important for targeting of CR educational offerings. Finally, identification of key CR skills and abilities might facilitate a redesign of GME educational offerings to ensure that Non-CR residents have adequate opportunities to develop such skills.

### Conflict of Interest

The authors declare no conflict of interest.

## Supplementary Material

Appendix 1

## References

[ref-2494] Lim R.F., Schwartz E., Servis M., Cox P.D., Lai A., Hales R.E. (2009). The chief resident in psychiatry: Roles and responsibilities. Acad Psychiat.

[ref-2495] Szymczak J.E., Bosk C.L. (2012). Training for efficiency: Work, time, and systems-based practice in medical residency. J Health Soc Behav.

[ref-2496] Wagner R., Weiss K.B., Passiment M.L., Nasca T.J. (2016). Pursuing excellence in clinical learning environments. J Grad Med Educ.

[ref-2497] Luciano G., Blanchard R., Hinchey K. (2013). Building chief residents' leadership skills. Medic Educ.

[ref-2498] Saxena A., Garg A., Desanghere L. (2015). Common pitfalls in the chief resident role: Impact on effective leadership practices. Intl J Leader Educ.

[ref-2499] Stoller J.K. (2009). Developing physician-leaders: A call to action. J Gen Intern Med.

[ref-2500] Gisondi M.A., Bavishi A., Burns J., Adler M.D., Wayne D.B., Goldstein J.L. (2017). Use of a chief resident retreat to develop key leadership skills. Medic Sci Educat.

[ref-2501] Pettit J.E., Wilson M.C. (2014). Leadership certificate program for chief residents. Medic Educat Develop.

[ref-2502] Clark J., Armit K. (2010). Leadership competency for doctors: A framework. Leader Health Serv.

[ref-2503] Hinchey K.T. (2008). A Textbook for Today's Chief Medical Resident.

[ref-2504] Jarousse L.A. (2010). Leadership in the era of reform. Hosp Health Netw.

[ref-2505] Frich J.C., Brewster A.L., Cherlin E.J., Bradley E.H. (2015). Leadership development programs for physicians: A systematic review. J Gen Intern Med.

[ref-2506] Moore J.M., Wininger D.A., Martin B. (2016). Leadership for all: An internal medicine residency leadership development program.

[ref-2507] Furnham A. (2005). Gender and personality differences in self- and other ratings of business intelligence. British Journal of Management.

[ref-2508] Gurian M., Annis B. (2008). Leadership and the sexes: using gender science to create success in business..

[ref-2509] Shambaugh R. (2013). Integrated leadership: Leveraging gender strengths to drive better business outcomes. Leader to Leader.

[ref-2510] Sinclair A. (1998). Doing leadership differently: gender, power, and sexuality in a changing business culture..

[ref-2511] Blumenthal D.M., Bernard K., Bohnen J., Bohmer R. (2014). Addressing the leadership gap in medicine: Residentsʼ need for systematic leadership development training.

[ref-2512] Kuo A.K., Thyne S.M., Chen H.C., West D.C., Kamei R.K. (2010). An innovative residency program designed to develop leaders to improve the health of children.

[ref-2513] Burk-Rafel J., Jones R.L., Farlow J.L. (2017). Engaging learners to advance medical education. Acad Medic.

[ref-2514] Hartzell J.D. (2007). Adult learning theory in medical education. American Journal of Medicine.

[ref-2515] Merriam S.B., Bierema L.L. (2014). Adult learning: Linking theory and practice.

[ref-2516] Michigan State University College of Osteopathic Medicine.

[ref-2517] https://www.surveymonkey.com/.

[ref-2518] Santric Milicevic M.M., Bjegovic-Mikanovic V.M., Terzic-Supic Z.J., Vasic V. (2010). Competencies gap of management teams in primary health care. Europ J Publ Health.

[ref-2519] IBM Corp Released 2017. IBM SPSS Statistics for Windows, Version 25.0.

[ref-2520] Minter R.M., Gruppen L.D., Napolitano K.S., Gauger P.G. (2005). Gender differences in the self-assessment of surgical residents. Amer J Surg.

[ref-2524] Mountford J., Webb C. (2009). When clinicians lead. Heath Leader Rev.

[ref-2523] Remler D.K., VanRyzin G.G. (2011). Research methods in practice: Strategies for description and causation..

