# Supplementary material for: Chief Resident Skills: A Study on Resident Perceptions of Skill Importance and Confidence
Source: Spartan Med Res J. 2018 Apr 27;3(1):6466. doi: 10.51894/001c.6466 (PMC7746022; doi:10.51894/001c.6466)
Supplement: Appendix 1 [file smrj_2018_3_1_6466_16928.pdf]

# Survey Instrument

## Statewide Campus System Chief Resident Learning Needs and Preferences Survey

### *Demographics*

First, please tell us about yourself:

1. Are you an intern, resident, or fellow in a Statewide Campus System/MSUCOM-affiliated training program?

- ◯ Yes
- ◯ No

2. Are you CURRENTLY a chief or co-chief Resident?

- ◯ Yes
- ◯ No

*Demographics continued...*

3. Gender

- ◯ Male
- ◯ Female
- ◯ Other

4. Degree Type

- ◯ M.D.
- ◯ D.O.

5. What is your current year in training (2016-2017 academic year)?

- ◯ PGY1
- ◯ PGY2
- ◯ PGY3
- ◯ PGY4
- ◯ PGY5
- ◯ Fellow
- ◯ Other (please specify)

6. Residency or Fellowship Program (Please enter your specialty discipline only):

7. Approximately how many residents are in your residency program (2016-17 AY)?

## Leadership

**Please use the slider to select a range of 1-5, with 1 the least and 5 the greatest.**

8a. How confident are you with your ability to lead multidisciplinary health care teams?

1 (not at all confident) 3 (confident) 5 (extremely confident)

8b. How important is leadership of multidisciplinary healthcare teams as a skill all chief residents need to have?

1 (not at all important) 3 (important) 5 (extremely important)

8c. What do you believe is the primary way residents learn or acquire skills in leadership of multidisciplinary health care teams?

- Formal classroom lecture from expert(s)
- Independent reading/researching the topic
- Trial and error (learning by doing, and by reflective learning from mistakes)
- Observing and emulating faculty members and/or senior peers
- Talking with others, asking questions of peers and/or faculty members

Other (please specify)

9a. How confident are you with leading quality improvement projects?

1 (not at all confident) 3 (confident) 5 (extremely confident)

9b. How important is leading quality improvement projects as a skill or ability all chief residents need to have?

1 (not at all important) 3 (important) 5 (extremely important)

9c. What do you believe is the primary way residents learn or acquire skills in leading quality improvement projects?

- ◯ Formal classroom lecture from expert(s)
- ◯ Independent reading/researching the topic
- ◯ Trial and error (learning by doing, and by reflective learning from mistakes)
- ◯ Observing and emulating faculty members and/or senior peers
- ◯ Talking with others, asking questions of peers and/or faculty members

Other (please specify)

[ ]

10a. How confident are you with understanding leadership and communication styles of self and others?

1 (not at all confident) | 3 (confident) | 5 (extremely confident)

10b. How important is understanding leadership and communication styles of self and others as a skill all chief residents need to have?

1 (not at all important) | 3 (important) | 5 (extremely important)

10c. What do you believe is the primary way residents learn or acquire skills in understanding leadership and communication styles of self and others?

- ◯ Formal classroom lecture from expert(s)
- ◯ Independent reading/researching the topic
- ◯ Trial and error (learning by doing, and by reflective learning from mistakes)
- ◯ Observing and emulating faculty members and/or senior peers
- ◯ Talking with others, asking questions of peers and/or faculty members

Other (please specify)

[ ]

11a. How confident are you with using strategies to facilitate the well-being of self and others, including fatigue and stress management/mitigation?

1 (not at all confident) | 3 (confident) | 5 (extremely confident)

11b. How important are understanding strategies for facilitating the well-being of self and others, including fatigue and stress management/mitigation, as a skill or ability all chief residents need to have?

1 (not at all important) 3 (important) 5 (extremely important)

11c. What do you believe is the primary way residents learn or acquire skills in strategies for facilitating the well-being of self and others, including fatigue and stress management/mitigation?

- Formal classroom lecture from expert(s)
- Independent reading/researching the topic
- Trial and error (learning by doing, and by reflective learning from mistakes)
- Observing and emulating faculty members and/or senior peers
- Talking with others, asking questions of peers and/or faculty members

Other (please specify)

Statewide Campus System Chief Resident Learning Needs and Preferences Survey

## Teaching

**Please use the slider to select a range of 1-5, with 1 the least and 5 the greatest.**

12a. How confident are you in giving effective feedback?

1 (not at all confident) | 3 (confident) | 5 (extremely confident)

12b. How important is giving effective feedback as a skill or ability all chief residents need to have?

1 (not at all important) | 3 (important) | 5 (extremely important)

12c. What do you believe is the primary way residents learn or acquire skills in giving effective feedback?

- Formal classroom lecture from expert(s)
- Independent reading/researching the topic
- Trial and error (learning by doing, and by reflective learning from mistakes)
- Observing and emulating faculty members and/or senior peers
- Talking with others, asking questions of peers and/or faculty members

Other (please specify)

13a. How confident are you with planning and conducting educational sessions such as didactics or journal club?

1 (not at all confident) | 3 (confident) | 5 (extremely confident)

13b. How important are planning and conducting educational sessions such as didactics or journal clubs as a skill or ability all chief residents need to have?

1 (not at all important) | 3 (important) | 5 (extremely important)

13c. What do you believe is the primary way residents learn or acquire skills in planning and conducting educational sessions such as didactics or journal club?

- ◯ Formal classroom lecture from expert(s)
- ◯ Independent reading/researching the topic
- ◯ Trial and error (learning by doing, and by reflective learning from mistakes)
- ◯ Observing and emulating faculty members and/or senior peers
- ◯ Talking with others, asking questions of peers and/or faculty members

Other (please specify)

______________________

14a. How confident are you with your skills in mentoring & coaching junior peers and medical students?

| 1 (not at all confident) | 3 (confident) | 5 (extremely confident) |
|---|---|---|

14b. How important are mentoring and coaching strategies as skills all chief residents need to have?

| 1 (not at all important) | 3 (important) | 5 (extremely important) |
|---|---|---|

14c. What do you believe is the primary way residents learn or acquire skills in mentoring & coaching?

- ◯ Formal classroom lecture from expert(s)
- ◯ Independent reading/researching the topic
- ◯ Trial and error (learning by doing, and by reflective learning from mistakes)
- ◯ Observing and emulating faculty members and/or senior peers
- ◯ Talking with others, asking questions of peers and/or faculty members

Other (please specify)

______________________

15a. How confident are you with using effective teaching strategies and practices?

| 1 (not at all confident) | 3 (confident) | 5 (extremely confident) |
|---|---|---|

15b. How important are effective teaching strategies and practices as skills or abilities chief residents need to have?

1 (not at all important) 3 (important) 5 (extremely important)

15c. What do you believe is the primary way residents learn or acquire skills in teaching strategies and practices?

- Formal classroom lecture from expert(s)
- Independent reading/researching the topic
- Trial and error (learning by doing, and by reflective learning from mistakes)
- Observing and emulating faculty members and/or senior peers
- Talking with others, asking questions of peers and/or faculty members

Other (please specify)

## Time management

Pleaseusetheslidertoselectarangeof1-5,with1theleastand5thegreatest.

16a. How confident are you with communication strategies for managing conflict?

1 (not at all confident) 3 (confident) 5 (extremely confident)

16b. How important are communication strategies for managing conflict as a skill all chief residents need to have?

1 (not at all important) 3 (important) 5 (extremely important)

16c. What do you believe is the primary way residents learn or acquire skills in managing conflict?

- Formal classroom lecture from experts
- Independent reading/researching the topic
- Trial and error (learning by doing, and by reflective learning from mistakes)
- Observing and emulating faculty members and/or senior peers
- Talking with others, asking questions of peers and/or faculty members

Other (please specify)

17a. How confident are you with administrative management responsibilities such as scheduling, directing work flow, and managing disciplinary issues?

1 (not at all confident) 3 (confident) 5 (extremely confident)

17b. How important are administrative management responsibilities such as scheduling, directing work flow, and managing disciplinary issues, as a skill all chief residents need to have?

1 (not at all important) 3 (important) 5 (extremely important)

17c. What do you believe is the primary way residents learn or acquire skills in administrative management responsibilities such as scheduling, directing work flow, and managing disciplinary issues?

- ◯ Formal classroom lecture from experts
- ◯ Independent reading/researching the topic
- ◯ Trial and error (learning by doing, and by reflective learning from mistakes)
- ◯ Observing and emulating faculty members and/or senior peers
- ◯ Talking with others, asking questions of peers and/or faculty members

Other (please specify)

18a. How confident are you with your skills in time management and efficiency (ability to prioritize multiple demands with limited time)?

1 (not at all confident) 3 (confident) 5 (extremely confident)

18b. How important is time management and efficiency as a skill all chief residents need to have?

1 (not at all important) 3 (important) 5 (extremely important)

18c. What do you believe is the primary way residents learn or acquire skills in time management and efficiency?

- ◯ Formal classroom lecture from experts
- ◯ Independent reading/researching the topic
- ◯ Trial and error (learning by doing, and by reflective learning from mistakes)
- ◯ Observing and emulating faculty members and/or senior peers
- ◯ Talking with others, asking questions of peers and/or faculty members

Other (please specify)

## Statewide Campus System Chief Resident Learning Needs and Preferences Survey

### *Disqualification page*

At this time we are only looking for information pertaining to current residents in a Statewide Campus System/MSUCOM -affiliated residency program.

Thank you for your time.

## Survey Complete!

**Thank you for your time.**
